# Prevalence of complex post-traumatic stress disorder in refugees and asylum seekers: systematic review

**DOI:** 10.1192/bjo.2021.1013

**Published:** 2021-10-15

**Authors:** Umanga de Silva, Naomi Glover, Cornelius Katona

**Affiliations:** Division of Psychiatry, University College London, UK; Division of Psychiatry, University College London, UK; Division of Psychiatry, University College London, and Helen Bamber Foundation, UK

**Keywords:** Complex post-traumatic stress disorder, refugees, forced displacement, complex trauma, systematic review

## Abstract

**Background:**

Refugees and asylum seekers often report having experienced numerous complex traumas. It is important to understand the prevalence of complex post-traumatic stress disorder (CPTSD), which can follow complex traumas.

**Aims:**

This systematic review aims to summarise the available literature reporting the prevalence in refugees and asylum seekers of three operationalised definitions of CPTSD: the ICD-11 diagnostic criteria, the ICD-10 criteria (for enduring personality change after catastrophic experience) and the DSM-IV criteria (for disorders of extreme stress not otherwise specified).

**Method:**

Six electronic databases were searched for studies reporting the prevalence of CPTSD in adult refugee and/or asylum-seeking samples. Owing to heterogeneity between the studies, a narrative synthesis approach was used to summarise studies. Methodological quality was assessed using the Joanna Briggs Critical Appraisal Checklist for Prevalence Studies. This systematic review has been registered with PROSPERO (registration number CRD42020188422, https://www.crd.york.ac.uk/prospero/display_record.php?RecordID=188422).

**Results:**

Systematic searches identified 15 eligible studies, with 10 examining treatment-seeking samples and five using population samples. CPTSD prevalence in treatment-seeking samples was between 16 and 38%. Prevalence in population samples ranged from 2.2 to 9.3% in four studies, with the fifth reporting a much higher estimate (50.9%).

**Conclusions:**

This review highlights both the high prevalence of CPTSD in treatment samples and the lack of research aiming to establish prevalence of CPTSD in refugee and asylum-seeking populations. Understanding the prevalence of these disabling disorders has implications for policy and healthcare services for the appropriate promotion, planning and provision of suitable treatment and interventions for this highly traumatised population.

## Refugee crisis

Approximately 79.5 million individuals worldwide have been forcibly displaced.^1^ Of these, 45.7 million have been displaced internally, 29.6 million have been externally displaced and 4.2 million are seeking asylum. Forced displacement typically follows human rights violations, conflict, persecution or events causing serious public disorder, with refugees and asylum seekers experiencing ‘complex traumas’.

## Complex trauma

Complex traumas are characteristically interpersonal in nature (i.e. are perpetrated deliberately by other people) and involve repeated prolonged and often multiple traumatic exposures between victim and perpetrator(s). The victim has typically been held in conditions from which they are unable to escape, because of physical, maturational, social, family/environmental or psychological constraints.^2^ Refugees and asylum seekers often report experiencing direct exposure to multiple types of complex traumatic experiences,^3^ including sexual violence, torture, imprisonment, enforced isolation, physical assault, and the murder of friends or family members.^4^ In addition to pre-migration traumas experienced in the individuals’ countries of origin, traumatic events can also be experienced during forced migration journeys,^5^^,^^6^ for instance, at the hands of human smugglers or traffickers or in refugee camps. This can result in trauma-related mental health disorders.^7^

## Complex post-traumatic stress disorder

In response to the observation that complex trauma can lead to mental health sequalae not adequately captured by prevailing diagnostic formulations of post-traumatic stress disorder (PTSD), Herman^2^ in 1992 proposed the term complex PTSD (CPTSD). PTSD typically follows single traumatic events that need not be interpersonally caused, such as natural disasters and accidents.^8^ However, individuals with experiences of complex trauma (particularly in the context of coercive control) may experience symptoms more varied and severe than the intrusion, avoidance and hyper-arousal symptoms classically associated with PTSD.

Enduring personality change after catastrophic events (EPCACE) was included in the ICD-10^9^ to capture the personality changes which can follow catastrophic stressors. Single traumatic events not interpersonal in nature are excluded as possible catastrophic stressors. A diagnosis of EPCACE requires personality changes attributed to trauma to last for a minimum of 2 years and can involve a mistrustful or hostile attitude towards the world, social withdrawal, persistently feeling hopeless or empty, estrangement and continuously feeling on edge.^9^ The criteria have been criticised for being overinclusive, with this diagnosis underutilised in both research and clinical settings.^10^

The DSM-IV^11^ included an equivalent symptom group referred to as disorder of extreme stress not otherwise specified (DESNOS). Core features of DESNOS include changes in sustaining beliefs, impulse and affect regulation, somatisation, self-perception, relationships with others and dissociation. DESNOS was viewed only as a symptom group within PTSD, as field trials indicated that almost all participants meeting DESNOS criteria also met PTSD criteria.^12^

The diagnostic category of CPTSD has been incorporated into the ICD-11^13^ as a separate disorder to PTSD. It consists of symptom clusters involving disturbances in self-organisation (DSO) in addition to the classical PTSD clusters of intrusion, avoidance, and hyper-arousal. The DSO symptom clusters include affective dysregulation, disturbances in relational function and negative self-concept. In addition, CPTSD requires endorsing additional symptoms indicating functional impairment. CPTSD reflects the depletion of social, psychological and emotional resources resulting from protracted conditions of trauma,^14^ leading to greater functional impairment than PTSD.

**Fig. 1:**
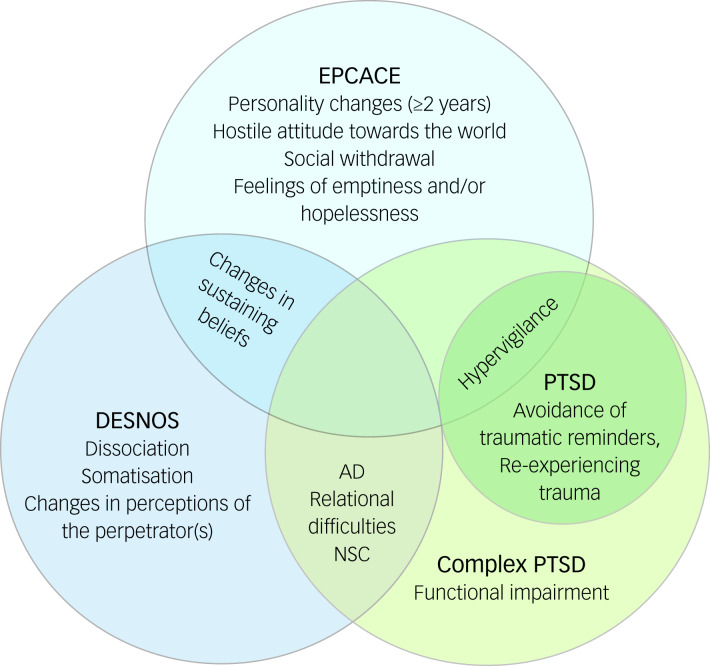
Overlaps and differences among definitions of enduring personality change after catastrophic events (EPCACE), disorder of extreme stress not otherwise specified (DESNOS), complex post-traumatic stress disorder (PTSD) and ICD-11 PTSD. AD, affective dysregulation; NSC, negative self-concept.

CPTSD, EPCACE and DESNOS have some overlapping symptoms. All three attempt to capture the more varied mental health sequalae that follow complex trauma. However, the diagnostic conceptualisations are not identical (Fig. 1). Unlike DESNOS, ICD-11 CPTSD is a diagnostic category distinct from PTSD, and unlike EPCACE, CPTSD criteria do not include personality change.

The applicability of CPTSD symptomatology has been demonstrated in various trauma samples, including individuals who have suffered childhood physical or sexual abuse,^14^ former prisoners of war^15^ and institutional abuse survivors.^16^ Although refugees and asylum seekers are a highly traumatised population, less is known about the prevalence of CPTSD in this population. The increase in the number of forcibly displaced people requiring resettlement in recent years requires physicians and health service providers to be aware of the mental disorders prevalent in this population. Treatment needs for PTSD and DSO symptoms may differ; research indicates that solely trauma-focused treatment can overwhelm those who have suffered complex trauma,^17^ with a phased treatment approach required instead.^18^ An understanding of the current prevalence of CPTSD in this population (and the associated treatment implications) is therefore highly important.

ter Heide et al^18^ have reported findings of an unpublished systematic review of five studies that used validated measures to assess the prevalence of DESNOS among refugees. The review incorporated two non-treatment-seeking and three treatment-seeking refugee populations displaced internally and externally. Relative to a diagnosis of DESNOS, no diagnosis or a PTSD diagnosis was more likely. They concluded that the more severe DESNOS symptoms should not be assumed to occur in a population because of the presence of complex traumatic experiences. However, the authors acknowledged that their conclusions were based on the limited evidence available at the time.^18^ This review also pre-dated the introduction of ICD-11 CPTSD, and there has since been an expansion in the literature examining this disorder among refugee and asylum-seeking populations.

A recent systematic review by Mellor et al^19^ also reported the prevalence of CPTSD, DESNOS and EPCACE in samples of refugees and displaced individuals, and further examined predictors of CPTSD and the associations between CPTSD and common mental disorders. The results demonstrated a wide range of CPTSD prevalence among studies, with authors positing this to reflect geographic and contextual factors.^19^ However, not all of the included studies examining CPTSD followed ICD-11 CPTSD diagnostic criteria or used diagnostic tools specific to ICD-11 CPTSD to diagnose participants. Furthermore, not all studies consisted of forcibly displaced samples. There remains the need for further synthesis of data on the prevalence of CPTSD, EPCACE and DESNOS among refugees and asylum seekers in treatment-seeking and population samples. An updated systematic review examining this is therefore timely.

## Aims and method

This systematic review provides an estimate of the prevalence of CPTSD among adult refugee and asylum-seeking samples. The term ‘refugees’ refers to individuals forcibly displaced internally within their country of origin or externally displaced in a foreign country.

The protocol was registered with the International Prospective Register of Systematic Reviews (PROSPERO; CRD42020188422, https://www.crd.york.ac.uk/prospero/display_record.php?RecordID=188422). The Preferred Reporting Items for Systematic Reviews and Meta-Analyses (PRISMA) checklist was followed^20^ (see Supplementary Appendix 3 available at https://doi.org/10.1192/bjo.2021.1013).

### Criteria for study inclusion

Eligible studies were required to include samples of refugees and/or asylum seekers of any ethnicity who were over 18 years of age at the time measures were administered. The use of validated or standardised diagnostic methods specific to the diagnoses of ICD-11 CPTSD, ICD-10 EPCACE or DSM-4 DESNOS to provide the corresponding diagnoses was also required, as was reporting of the prevalence of CPTSD, EPCACE or DESNOS within the refugee and/or asylum-seeking samples. As sampling biases are more likely in smaller samples,^21^ only studies with a sample size greater than 50 were considered eligible. When numerous articles reported the same data from the same sample, the earliest publication was used.

### Search strategy

The electronic databases PsychINFO, Embase, Medline, EBSCO and ProQuest PTSDpubs, a database specifically for PTSD and trauma literature, were searched from the time of their inception to 25 March 2020. A final search of these databases was conducted on 8 Jan 2021 to capture any studies published after the first search.

The search terms consisted of terms relating to refugees, asylum seekers, CPTSD, DESNOS and EPCACE. Full search strategies for all databases are provided in Supplementary Appendix 1. The reference lists of studies eligible for inclusion in the review were manually searched by the first author for further relevant studies not captured by the systematic searches. None were found.

### Study selection

The identification of studies followed procedures outlined by PRISMA.^22^ Titles and abstracts were initially screened by U.D.S. for articles deemed potentially relevant. Following this, all authors independently reviewed the full texts of each study to establish whether they were eligible for inclusion. All authors then discussed the final studies to be included.

### Data extraction

Data were first extracted on 3 July 2020 and recorded in a tabular form including sample setting; time spent in host country; mean age and age range; measurement tools used to assess CPTSD, EPCACE and DESNOS; measurement tools used to assess additional diagnoses; diagnostic criteria used for diagnosing CPTSD, EPCACE and DESNOS, and additional disorders assessed; and the prevalence of CPTSD, EPCACE and DESNOS, and other diagnoses assessed. Additional study characteristics extracted were gender breakdown, age statistics and duration spent in the host country.

### Synthesis and analysis of data

The heterogeneity among the study designs, methods, populations and the reported effect estimates in the eligible studies did not allow for meta-analysis. Consequently, a narrative synthesis was conducted to synthesise findings. The point prevalence of CPTSD, EPCACE and DESNOS among study samples was described and compared. Owing to the expected disparity in the prevalence estimates between treatment-seeking and population samples, these groupings are presented separately. Studies were also grouped by diagnostic system used. Where possible, the prevalence rates of the disorders of interest among refugee and asylum-seeking samples were compared with the prevalence of other diagnoses assessed within the same samples.

### Risk of bias assessments

The Joanna Briggs Institute Critical Appraisal Checklist for Prevalence Data^23^ was used to assess the methodological quality of each individual study. This considers nine domains: suitability of the sample frame; appropriateness of participant recruitment; sample size sufficiency; adequacy of the setting and participant description; adequacy in reporting the sample; validity of the diagnostic measures; suitability of the statistical analyses; and adequacy of the response rates. The majority view among all authors for each criterion was used.

## Results

### Overview of study selection

**Fig. 2:**
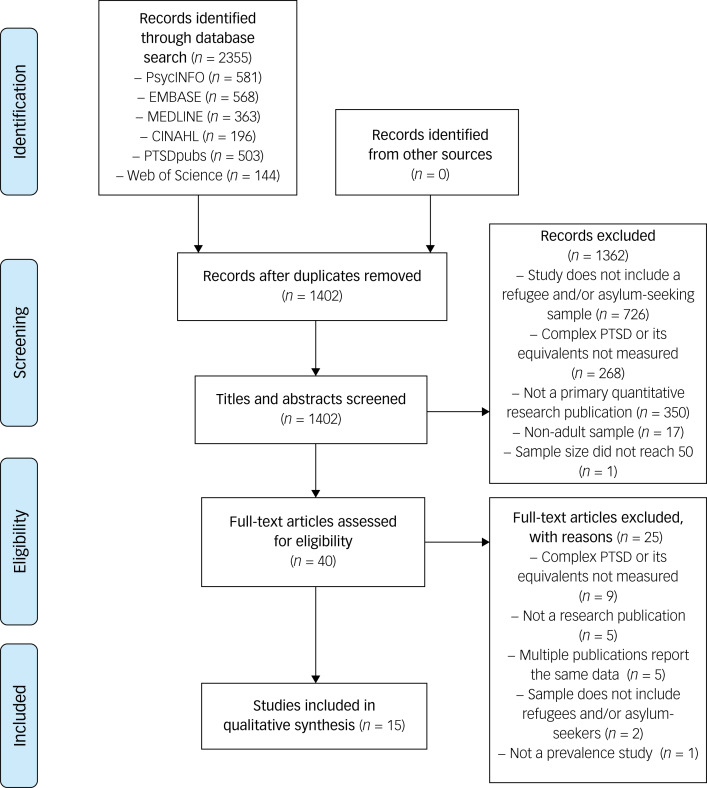
PRISMA flowchart depicting study selection based on searches conducted on 25 March 2020 and 8 January 2021. PTSD, post-traumatic stress disorder.

A flowchart of the study selection process is shown in Fig. 2. The systematic searches of the six electronic databases identified 2355 results, from which the titles and abstracts of 1402 studies were assessed for eligibility. Of these, 40 records were considered eligible for full-text screening. In the second stage of screening, 25 records were excluded. Fifteen studies were found to be eligible and were included in the review, 14 of which were found in the searches conducted on 25 March 2020, with the remaining study found in the second search on 8 January 2021. Of the included studies, ten assessed CPTSD, three assessed DESNOS and two assessed EPCACE.

Study characteristics are detailed in Supplementary Appendix 2. The mean sample size per study was 258.4 (range 61–1200), with a total sample size of 3876 measuring CPTSD. All studies were conducted in the countries hosting the refugees. Eight were conducted in Europe, two in the Middle East, three in Oceania, one in Africa and one in the USA. The time spent in the host country at the time measures were administered varied within and between samples, with means ranging from 11.1 months to 27 years across studies. Data for time spent in the host country were unavailable for seven studies.

### Prevalence of CPTSD in treatment-seeking samples

**Fig. 3:**
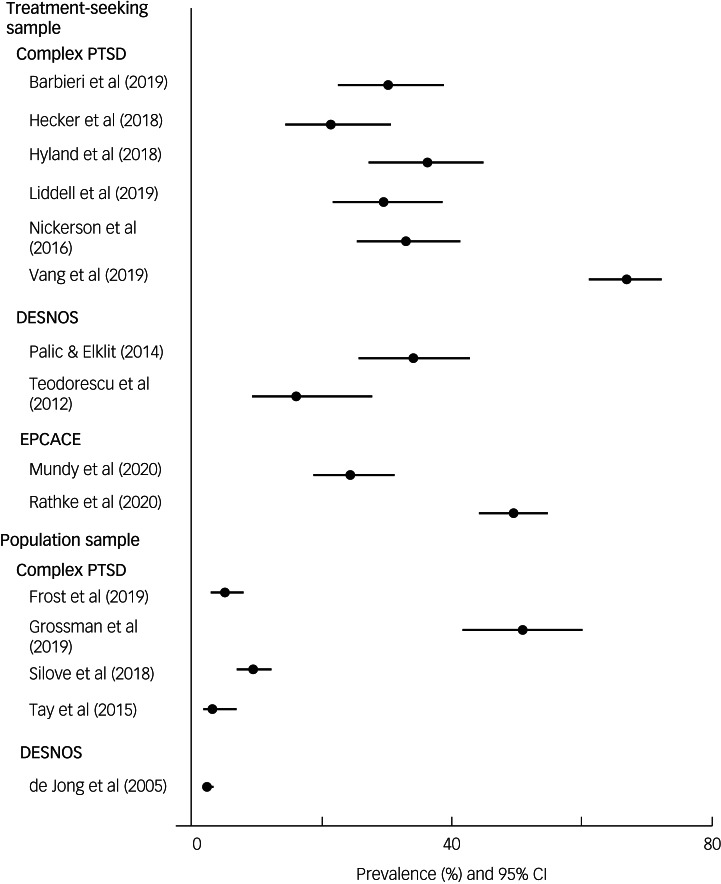
Prevalence of complex post-traumatic stress disorder (PTSD), enduring personality change after catastrophic events (EPCACE) and disorder of extreme stress not otherwise specified (DESNOS) in treatment-seeking and population samples.

Ten studies used treatment-seeking samples, with the prevalence of CPTSD, DESNOS or EPCACE ranging from 16 to 66.9% (Fig. 3). The point prevalence of CPTSD was measured in six studies (Table 1). A CPTSD prevalence of 21.3% was reported in a culturally diverse refugee sample resettled in Switzerland.^28^ In a sample of Syrian refugees living in Lebanon, a CPTSD prevalence of 36.1% was found,^29^ and a prevalence estimate of 66.9% was reported in a culturally diverse refugee sample in Denmark.^36^ One study^4^ investigating a culturally diverse sample of refugees resettled in Sydney, Australia, reported a CPTSD prevalence estimate of 29.5%. In a study investigating a sample of African refugees residing in Italy, a prevalence of 30% was reported.^24^ Nickerson et al^31^ reported a prevalence of 32.8% in a culturally diverse refugee and asylum-seeking sample in Switzerland.

**Table 1:** Point prevalence of complex post-traumatic stress disorder (CPTSD) in included studies

Publication	Sample size	CPTSD measurement tool	Diagnostic criteria	CPTSD prevalence	95% CI
Barbieri et al (2019)^24^	*n* = 120	Seven items from the PCL-5^40^ to represent DSO clusters, in accordance with the ICD-11 ITQ criteria. Self-report measure delivered with assistance of a cultural mediator, clinical psychologist, and medical doctor.	PTSD and scores of ≥6 on three AD (hyperactivation) items or of ≥2 on one AD item (deactivation); scores of ≥4 on 2 NSC items; and a score of ≥2 on one DR item.	30% (*n* = 36)	22.53 to 38.72
de Jong et al (2005)^25^	*n* = 1200	SIDES,^41^ administered and rated by trained local fieldworkers.	Meeting the clinical thresholds for the six domains of CPTSD.	2.2% (*n* = 26)	1.48 to 3.16
Frost et al (2019)^26^	*n* = 308	Items from the PTSD scale of the AUDADIS-IV^42^ as indicators of two DSO symptom clusters (AD and DR), and items from the low mood scale as indicators of the NSC cluster. Self-report measure administered in interviews conducted by trained laypersons.	In addition to meeting PTSD criteria, one of two symptoms from each DSO cluster was endorsed, with responses coded as 1 (symptom present) or 0 (symptom absent).	4.9% (*n* = 15)	2.97 to 7.88
Grossman et al (2019)^27^	*n* = 108	ITQ^14^ Self-report measure administered in interviews conducted by trained research assistants.	Fulfilment of PTSD criteria and endorsing one of two symptoms from every DSO cluster, signified by scores of ≥3.	50.9% (*n* = 55)	41.63 to 60.16
Hecker et al (2018)^28^	*n* = 94	ITQ^43^ Self-report measure administered in structured interviews or as guided questionnaire Assessments conducted by trained assessors.	Fulfilment of PTSD criteria, in addition to scores of ≥10 or ≥8, ≥8 and ≥6 for AD-hypoactivation or hyperactivation, NSC and DR, respectively.	21.3% (*n* = 20)	14.22 to 30.59
Hyland et al (2018)^29^	*n* = 110	ITQ (M. Cloitre, personal communication, 27 September 2021) Self-report measure administered by trained psychotherapists.	Fulfilment of PTSD criteria, in addition to scores of ≥10 or ≥8, ≥8 and ≥6 for AD-hypoactivation or hyperactivation, NSC and DR, respectively.	36.1% (*n* = 39)	27.14 to 44.75
Liddell et al (2019)^4^	*n* = 112	An initial version of the ITQ consisting of nine items assessing CPTSD symptoms, and a subsequent version of the ITQ^43^ consisting of 17 ITQ items for the three DSO clusters and more closely resembling the current instrument. Self-report measures completed in clinical interviews with trained research or clinical psychologists.	Fulfilment of PTSD criteria and meeting thresholds for DSO clusters.	29.5% (*n* = 33)	21.82 to 38.47
Mundy et al (2020)^30^	*n* = 182	ICD-10 EPCACE diagnostic criteria.^9^	Fulfilment of ICD-10 EPCACE criteria, assessed during pre-treatment clinical interviews by medical doctors assisted by trained interpreters when necessary.	24.2% (*n* = 44)	18.53 to 30.89
Nickerson et al (2016)^31^	*n* = 134	Assessed using a total of six items closely related to ICD-11 CPTSD criteria, with two taken from the DERS^44^ and the Hopkins Symptom Checklist,^45^ and one item from the ECR^46^ and the PDS.^49^ Self-report measure completed with assistance from a trained master's-level student, or by a psychiatrist or clinical psychologist.	Meeting PTSD criteria, in addition to endorsing at least one item from each DSO symptom cluster. Scores of ≥3, ≥3 and ≥4 for AD, NSC and DR items, respectively, indicated symptom endorsement.	32.8% (*n* = 44)	25.46 to 41.17
Palic & Elklit (2014)^32^	*n* = 116	SIDES-SR^47^ administered as a self-report measure.	Meeting the specified clinical thresholds for all six DESNOS symptom clusters.	34% (*n* = 39)	25.67 to 42.62
Rathke et al (2020)^33^	*n* = 330 (of a study sample size of 627)	ICD-10 EPCACE diagnostic criteria.^9^	EPCACE diagnoses made by medical doctors during routine assessments of ICD-10 criteria.	49.5% (*n* = 163)	44.04 to 54.76
Silove et al (2018)^34^	*n* = 487	R-MHAP^48^ Self-report measure administered in interviews conducted by trained field teams from the West Papuan community.	R-MHAP diagnostic criteria are based on the DSM-4, DSM-5, ICD-10 and ICD-11. Symptom endorsement required the highest two ratings from a four-point severity scale. A diagnosis of CPTSD required the additional fulfilment of PTSD criteria.	9.3% (*n* = 45)	6.98 to 12.14
Tay et al (2015)^35^	*n* = 230	R-MHAP^48^ Self-report measure completed in interviews conducted by trained West Papuan refugees.	Algorithms derived from definitions of CPTSD and PTSD in the DSM-4, DSM-5, ICD-10 and ICD-11 were used to provide diagnoses of CPTSD.	3% (*n* = 8)	1.77 to 6.71
Teodorescu et al (2012)^3^	*n* = 61	SIDES^41^ Structured interviews of the observer-rated measure conducted by one trained researcher.	Meeting the clinical thresholds for all seven domains of DESNOS: (a) changes in affect regulation; (b) dissociation; (c) changes in self-perception; (d) somatisation; (e) changes in relations with others; (f) changes in systems of meaning; and (g) changes in how the perpetrator is perceived.	16% (*n* = 10)	9.16 to 27.61
Vang et al (2019)^36^	*n* = 284	ITQ^43^ Self-report measure that was read aloud to 70.6% of participants by a trained clinician and translated by an interpreter.	A PTSD diagnosis, in addition to the endorsement of one item from every CPTSD cluster and an indicator of functional impairment, signified by scores of ≥2.	66.9% (*n* = 190)	61.24 to 72.12

PCL-5, PTSD Checklist for DSM-5 (PCL-5); DSO, disturbances in self-organisation; ITQ, International Trauma Questionnaire; AD, affective dysregulation; NSC, negative self-concept; DR, disturbances in relational function; SIDES, Structured Interview for Disorders of Extreme Stress; AUDADIS-IV, Alcohol Use Disorder and Associated Disabilities Interview Schedule-IV; EPCACE, enduring personality change after catastrophic events; DERS, Difficulties in Emotion Regulation Scale; ECR, Experiences in Close Relationships Scale; PDS, Posttraumatic Diagnostic Scale; SIDES-SR, Self-Report Inventory for Disorders of Extreme Stress; R-MHAP, Refugee-Mental Health Assessment Package.

Two studies reported prevalence estimates of DESNOS. In a culturally diverse sample of refugees in Norway, a point prevalence of 16% was reported.^3^ Another study looked at a sample of Bosnian former refugees in Denmark, finding a prevalence of 34%.^32^

Two studies measured EPCACE in samples of culturally diverse refugees resettled in Denmark, reporting point prevalence estimates of 49.5^33^ and 24.2%.^30^

### Prevalence of CPTSD in population samples

Five studies used population samples, with prevalence ranging from 2.2 to 50.9%. Four measured CPTSD. Grossman et al^27^ reported a prevalence of 50.9% in recently liberated female Yazidi captives resettled in camps in Kurdistan. In separate samples of West Papuan refugees residing in settlements in Papua New Guinea, prevalence rates of 9.3^34^ and 3%^35^ were reported. One study^26^ investigated a culturally diverse sample of refugees in the USA, reporting a prevalence of 4.9%.

One study measured the prevalence of DESNOS in a sample of Ethiopian refugees in Eritrean refugee shelters and found it to be 2.2%.^25^

EPCACE was not measured in any population sample of refugees and/or asylum seekers.

## Prevalence of additionally measured diagnoses relative to CPTSD, DESNOS and EPCACE

### Treatment-seeking samples

**Fig. 4:**
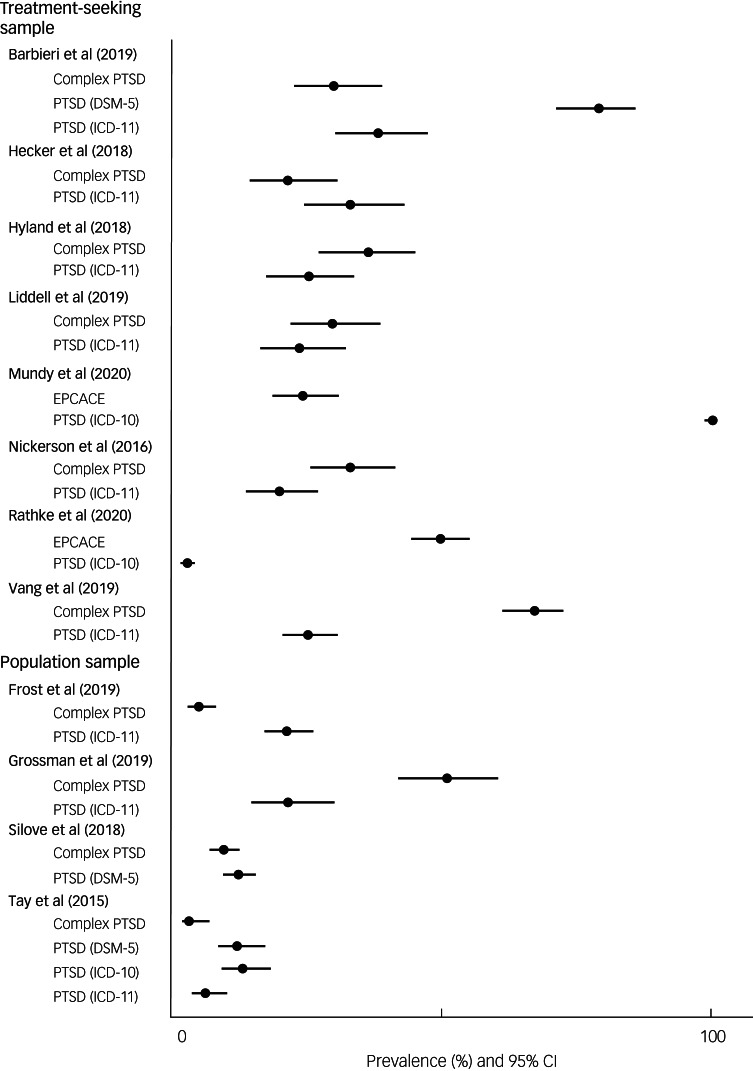
Prevalence of the most frequently measured additional diagnoses of DSM-5 post-traumatic stress disorder (PTSD) and ICD-11 PTSD compared with complex PTSD in treatment-seeking and population samples.

ICD-11 PTSD prevalence was measured in six studies (Fig. 4). Two reported higher ICD-11 PTSD prevalence (38 and 32.9%, respectively) compared with CPTSD (30 and 21.3%, respectively).^24^^,^^28^ Four studies reported lower ICD-11 PTSD prevalence estimates compared with CPTSD.^4^^,^^29^^,^^31^^,^^36^

One study measured DSM-5 PTSD, reporting a higher prevalence estimate of 79% compared with a CPTSD prevalence of 30%^24^. One study reported prevalence estimates of 82% for DSM-IV PTSD, 71% for major depressive disorder (MDD), 31% for generalised anxiety disorder (GAD), 41% for panic disorder, 49% for dysthymia, 0% for hypomania, 49% for agoraphobia, 49% for social phobia, 44% for obsessive–compulsive disorder, 10% for alcohol abuse disorder, 36% for nicotine dependence and 5% for bulimia, compared with 16% for DESNOS.^3^ Rathke et al reported prevalence estimates of 2.6% for ICD-10 PTSD, 49.5% for ICD-10 PTSD and comorbid EPCACE, 43.9% for ICD-10 PTSD with secondary psychotic features (PTSD-SP) and comorbid EPCACE, 25.8% for ICD-10 and comorbid depression, and 4.2% for ICD-10 PTSD-SP without comorbid depression, relative to 49.5% for EPCACE.^33^

One study^30^ reported a prevalence estimate of 93.8% for ICD-10 depression and 33.2% for other diagnoses, relative to a lower EPCACE prevalence estimate of 24.2%, in a sample of refugees with existing diagnoses of ICD-10 PTSD.

### Population samples

ICD-11 PTSD was measured in three studies, with the prevalence ranging between 6 and 21.3%. Two studies reported greater ICD-11 PTSD prevalence estimates (20.9 and 13%) relative to CPTSD (4.9 and 3%),^26^^,^^35^ with the remaining study reporting a lower PTSD prevalence estimate (21.3%) compared with CPTSD (50.9%).^27^ DSM-5 PTSD was assessed in two studies, with prevalence estimates of 12^35^ and 12.1%^34^ reported; DSM-5 PTSD prevalence estimates were higher than CPTSD prevalence in both studies. One study^35^ reported prevalence estimates of 13% for DSM-IV PTSD and 13% for ICD-10 PTSD, whereas the CPTSD prevalence was 3%. Another study^34^ reported prevalence estimates of 56.2% for MDD, 26.5% for GAD, 8% for panic disorder, 9.9% for persistent complex bereavement, 9.9% for separation anxiety disorder and 5.6% for intermittent explosive disorder, compared with 9.3% for CPTSD.

### Methodological quality

Risk of bias assessments for all studies are presented in Table 2. Two studies used samples drawn through random selection.^25^^,^^26^ Two studies attempted to sample the entire population of eligible refugees.^34^^,^^35^ One study used a consecutively drawn sample.^36^ The sampling methods in the remaining ten studies were unclear or non-random.

**Table 2:** Methodological quality of included studies assessed using the Joanna Briggs Institute Critical Appraisal Checklist for Prevalence Studies^23^

Question	Was the sample frame appropriate to address the target population?	Were study participants sampled in an appropriate way?	Was the sample size adequate?	Were the study subjects and the setting described in detail?	Was the data analysis conducted with sufficient coverage of the identified sample?	Were valid methods used for the identification of the condition?	Was the condition measured in a standard, reliable way for all participants?	Was there appropriate statistical analysis?	Was the response rate adequate, and if not, was the low response rate managed appropriately?
Barbieri et al (2019)^24^	N	N	N	Y	Y	Y	Y	N	U
de Jong et al (2005)^25^	Y	Y	Y	Y	Y	Y	Y	N	Y
Frost et al (2019)^26^	Y	Y	Y	Y	Y	Y	Y	N	U
Grossman et al (2019)^27^	Y	U	Y	Y	U	Y	Y	N	U
Hecker et al (2018)^28^	N	U	N	Y	Y	Y	Y	N	Y
Hyland et al (2018)^29^	Y	N	N	Y	N	Y	Y	N	U
Liddell et al (2019)^4^	N	U	N	Y	U	Y	N	N	U
Mundy et al (2020)^30^	N	N	N	N	U	Y	Y	N	N
Nickerson et al (2016)^31^	N	N	N	Y	Y	Y	Y	N	Y
Palic & Elklit (2014)^32^	Y	Y	N	Y	N	Y	Y	N	N
Rathke et al (2020)^33^	Y	U	N	Y	Y	Y	Y	N	Y
Silove et al (2018)^34^	Y	Y	Y	Y	Y	Y	Y	N	Y
Tay et al (2015)^35^	Y	Y	Y	Y	Y	Y	Y	N	Y
Teodorescu et al (2012)^3^	N	N	N	Y	Y	Y	Y	N	Y
Vang et al (2019)^36^	Y	N	N	Y	Y	Y	Y	N	U

Y, Yes; N, No; U, Unclear.

The samples used in two studies were drawn from census data of refugees living in selected settlements.^34^^,^^35^ One study^25^ drew its sample from a list of registered displaced individuals, one study^26^ used a subsample selected from a randomly drawn and nationally representative sample of US non-institutionalised adults, and one study^27^ drew its sample from post-liberation camps. The remaining ten studies drew samples from treatment centres.^3^^,^^4^^,^^24^^,^^28^^–^^33^^,^^36^

As they were not stated in any of the included studies, the required sample sizes for prevalence estimates for treatment-seeking and population samples were calculated using the following formula^37^: 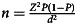, where *n* is the sample size, *Z* is the level of confidence statistic, *P* is the anticipated prevalence and *d* is the precision. Expected CPTSD prevalence estimates of 31.4% in treatment-seeking samples and 4.9% in population samples were assumed by calculating the median values of the reported prevalence estimates within these two sample types. Median values were used owing to the variability in prevalence estimates among studies, so as to reduce sensitivity to outliers. In addition, 95% confidence intervals and 5% precision were used in sample size calculations for both treatment-seeking and population samples. Required sample sizes for treatment-seeking and population samples were 331 and 72, respectively. Thus, sample sizes were insufficient in all ten studies with treatment-seeking samples but were sufficient for studies with population samples.

Two studies developed measures consisting of items from several scales^26^^,^^31^ but did not validate them. One used items representing DSO clusters from a PTSD scale but reported good internal consistency (Cronbach's α = 0.81).^24^ Another used unvalidated precursors to the International Trauma Questionnaire but reported good and high internal consistency for both within the sample (Cronbach's α = 0.75 and 0.92).^4^

## Discussion

### Interpretation of findings

Considering the sizeable and increasing population of refugees and asylum-seekers, studies reporting the prevalence of CPTSD, and previously EPCACE and DESNOS, are surprisingly scarce. Nevertheless, important conclusions can still be drawn from the studies identified in this review. Consistent with previous systematic reviews of studies examining the prevalence of CPTSD, EPCACE and DESNOS in refugees and asylum seekers,^18^^,^^19^ the results demonstrate that treatment-seeking samples generally report higher prevalence estimates of CPTSD, EPCACE and DESNOS relative to population samples. Individuals who have more severe symptoms may be more likely to seek treatment. However, one study with a population sample of recently liberated Yazidi women^27^ was an outlier, with the second highest prevalence estimate reported of all the included studies. Although determining the reason for this was not within the scope of this review, it may have been due to the high severity of the traumas experienced by this sample. The Yazidi women in this sample experienced repeated torture, sexual assault and sexual slavery during prolonged captivity; these experiences are closely associated with the specified risk factors of complex trauma. The homogeneity of this sample and the severity of trauma may contribute to a higher CPTSD prevalence.

Wide variations exist in the prevalence of CPTSD, EPCACE and DESNOS in the included studies. Potential contributing factors may include the geographic location of study implementation and culturally heterogenous study samples,^3^^,^^4^^,^^24^^,^^26^^,^^28^^,^^30^^,^^31^^,^^33^^,^^36^ as hypothesised by Mellor et al.^19^ Cultural heterogeneity within samples may be associated with differences in traumatic exposure, with greater experiences of CPTSD risk factors leading to a higher prevalence of CPTSD. However, heterogeneity in the reporting of sample demographics, risk factors and trauma exposure meant that a closer examination of this was beyond the scope of the current review.

The higher prevalence of CPTSD compared with PTSD in six of 13 studies reporting PTSD data highlights that the diagnostic criteria for PTSD may not capture the complex psychological responses that follow complex trauma. However, the data suggest that only a minority of refugees develop CPTSD.

Although self-report measures may overestimate prevalence,^38^ they were not consistently associated with higher prevalence estimates compared with observer- or clinician-rated measures. However, only three studies used observer-rated measures.

Differences in demographic characteristics may underpin the diversity in CPTSD prevalence. For instance, the length of time refugees spent in the host country, which is a predictor of the severity of CPTSD symptoms,^24^ varied greatly across studies. However, owing to inconsistencies in or the absence of reported post-migration stressors, it is unclear how prevalence estimates in the studies were affected.

### Strengths and limitations

This review is the first to examine the prevalence of CPTSD, EPCACE and DESNOS in refugees and asylum seekers. Independent screening of full-text studies by all three authors ensured consistency and the appropriateness of included studies. Risk of bias was assessed systematically.

The evidence presented is limited by the methodological constraints of the studies included. Although the studies reported data relevant to the review question, they were primarily aiming to examine the validity and structure of the ICD-11 CPTSD construct in refugee samples rather than to measure prevalence. The sampling frames were often inappropriate, with few studies using census data. Most samples were also non-randomly drawn. Based on calculations in this review, sample sizes were inadequate in all studies with treatment-seeking samples; this was further emphasised by the wide confidence interval for prevalence estimates. There is also a risk of non-response bias, as response rates and information regarding differences between respondents and non-respondents were infrequently reported.

Several studies developed their own measures from scales not designed to identify CPTSD. This may have resulted in measurement bias.

Although the terms CPTSD, EPCACE and DESNOS are often used interchangeably when discussing the sequelae of complex trauma, conceptual issues may exist in the use of all three diagnoses in this review. However, considering the scarcity of research on the prevalence of each of these three diagnoses among refugees and asylum seekers, including all three diagnoses can be considered useful in understanding the proportion of this population experiencing complex psychological responses to trauma.

### Implications

This systematic review has implications for treatment planning, highlighting the need to consider treatment needs among refugees and asylum seekers. Given that complex psychological responses are present in this population, awareness of prevalence estimates would facilitate the planning and provision of appropriate treatments targeting symptoms and encourage research to find safe treatment approaches that do not further traumatise these individuals. The addition of CPTSD as a diagnosis and the awareness of its prevalence among treatment-seeking refugees and asylum seekers may facilitate recognition of this disorder and ensure that adequate treatment is provided. Policy planners should also aim to promote mental health in this population, for instance, by addressing post-migration needs that may further prevent recovery from complex traumas.

### Future directions

This review highlights the gaps in the existing literature, demonstrating the clear need for methodologically robust epidemiological studies seeking to better understand the prevalence of CPTSD among refugees and asylum seekers. Studies should be representative of diverse refugee and asylum-seeking populations and include a range of cultures and trauma experiences. This would enable a more detailed explanation of factors underlying the variability of CPTSD prevalence estimates across the studies identified in this review, without any potential methodological issues influencing the results and conclusions drawn. Such studies would allow for more precise and generalisable estimates of the prevalence of this disabling disorder. This review could be used as a platform to encourage the planning of research to address this treatment gap.

This review highlighted several key methodological issues that should be considered in the design and implementation of future studies to ensure data reliability. There is a clear need for future studies examining treatment-seeking samples to satisfy sample size requirements. Furthermore, heterogeneity exists in the CPTSD measurement tools used in studies, both validated and unvalidated. Future studies should consider the validity of tools administered to facilitate a CPTSD diagnosis, and whether tools measure all CPTSD domains. This is particularly relevant when measuring CPTSD cross-culturally, where translated tools and instruments created specifically for a study, whether validated or not, may lack sufficient construct equivalence. Cultural nuances must be reflected in measurement tools to capture CPTSD within communities. The cultural salience of CPTSD cross-culturally should also be considered. It would be of interest for future studies to further examine risk and protective factors experienced prior to, during and following forced migration that can predict the development of CPTSD. This may also relate to diversity in sample demographics, where heterogeneity in factors such as the country of origin or resettlement potentially affect the type, severity and number of traumas experienced, which in turn may influence CPTSD prevalence.

Studies are also needed to measure the prevalence in this population of disorders with features overlapping with those of CPTSD. These include, for instance, borderline personality disorder, which shares the core feature of emotional dysregulation in a comparable way.^39^ Although patterns of response differ between CPTSD and these disorders, knowledge of the prevalence estimates of similar disorders occurring in this population would aid differential diagnosis and facilitate the provision of appropriate interventions and treatments to improve the mental health of refugees and asylum seekers suffering from disabling disorders such as CPTSD.

Future research should also examine the types of trauma which may be associated with CPTSD and the influence of post-migration factors, in order to aid diagnosis and appropriate treatment of traumatised asylum seekers and refugees and thereby promote their sustained recovery.

## Data Availability

The data supporting the findings of this review are available upon request from the corresponding author, U.D.S.

## Supplementary material

For supplementary material accompanying this paper visit http://dx.doi.org/10.1192/bjo.2021.1013. click here to view supplementary material

## References

[1] United Nations High Commissioner for Refugees. Global Trends: Forced Displacement in 2019. UNHCR, 2020 (https://www.unhcr.org/uk/statistics/unhcrstats/5ee200e37/unhcr-global-trends-2019.html).

[2] Herman JL. Complex PTSD: a syndrome in survivors of prolonged and repeated trauma. J Trauma Stress 1992; 5: 377–91.

[3] Teodorescu D-S, Heir T, Hauff E, Wentzel-Larsen T, Lien L. Mental health problems and post-migration stress among multi-traumatized refugees attending outpatient clinics upon resettlement to Norway. Scand J Psychol 2012; 53(4): 316–32. 22612589 10.1111/j.1467-9450.2012.00954.x

[4] Liddell BJ, Nickerson A, Felmingham KL, Malhi GS, Cheung J, Den M, Complex posttraumatic stress disorder symptom profiles in traumatized refugees. J Trauma Stress 2019; 32(6): 822–32. 31648412 10.1002/jts.22453

[5] Beiser M. Longitudinal research to promote effective refugee resettlement. Transcult Psychiatry 2006; 43(1): 56–71. 16671392 10.1177/1363461506061757

[6] Phillimore J. Refugees, acculturation strategies, stress and integration. J Soc Policy 2010; 40(3): 575–93.

[7] Bogic M, Njoku A, Priebe S. Long-term mental health of war-refugees: a systematic literature review. BMC Int Health Hum Rights 2015; 15(1): 29. 26510473 10.1186/s12914-015-0064-9 PMC4624599

[8] Breslau N, Davis GC, Andreski P, Peterson E. Traumatic events and posttraumatic stress disorder in an urban population of young adults. Arch Gen Psychiatry 1991; 48(3): 216–22. 1996917 10.1001/archpsyc.1991.01810270028003

[9] World Health Organization. International Statistical Classification of Diseases and Related Health Problems (10th Revision). WHO, 1992.

[10] Beltran RO, Silove D. Expert opinions about the ICD-10 category of enduring personality change after catastrophic experience. Compr Psychiatry 1999; 40(5): 396–403. 10509624 10.1016/s0010-440x(99)90147-5

[11] American Psychiatric Association. Diagnostic and Statistical Manual of Mental Disorders (4th edn). APA, 2000.

[12] Roth S, Newman E, Pelcovitz D, van der Kolk B, Mandel FS. Complex PTSD in victims exposed to sexual and physical abuse: results from the DSM-IV Field Trial for Posttraumatic Stress Disorder. J Trauma Stress 1997; 10(4): 539–55. 9391940 10.1023/a:1024837617768

[13] World Health Organization. International Statistical Classification of Diseases for Mortality and Morbidity Statistics (11th Revision). WHO, 2018.

[14] Cloitre M, Garvert DW, Brewin CR, Bryant RA, Maercker A. Evidence for proposed ICD-11 PTSD and complex PTSD: a latent profile analysis. Eur J Psychotraumatol 2013; 4(1): 20706. 10.3402/ejpt.v4i0.20706 PMC3656217 23687563

[15] Zerach G, Solomon Z. The relations between posttraumatic stress disorder symptoms and disorder of extreme stress (not otherwise specified) symptoms following war captivity. Isr J Psychiatr Relat Sci 2013; 50(3): 148–56. 24622473

[16] Knefel M, Garvert DW, Cloitre M, Lueger-Schuster B. Update to an evaluation of ICD-11 PTSD and complex PTSD criteria in a sample of adult survivors of childhood institutional abuse by Knefel & Lueger-Schuster (2013): a latent profile analysis. Eur J Psychotraumatol 2015; 6(1): 25290. 25557561 10.3402/ejpt.v6.25290 PMC4283031

[17] Herman JL. Trauma and Recovery. Basic Books, 1992.

[18] ter Heide FJJ, Mooren TM, Kleber RJ. Complex PTSD and phased treatment in refugees: a debate piece. Eur J Psychotraumatol 2016; 7(1): 28687. 26886486 10.3402/ejpt.v7.28687 PMC4756628

[19] Mellor R, Werner A, Moussa B, Mohsin M, Jayasuriya R, Tay AK. Prevalence, predictors and associations of complex post-traumatic stress disorder with common mental disorders in refugees and forcibly displaced populations: a systematic review. Eur J Psychotraumatol 2021; 12(1): 1863579. 10.1080/20008198.2020.1863579 PMC8725775 34992745

[20] Moher D, Liberati A, Tetzlaff J, Altman DG. Preferred reporting items for systematic reviews and meta-analyses: the PRISMA statement. PLoS Med 2009; 6(7): E1000097. 19621072 10.1371/journal.pmed.1000097 PMC2707599

[21] Fazel M, Wheeler J, Danesh J. Prevalence of serious mental disorder in 7000 refugees resettled in western countries: a systematic review. Lancet 2005; 365(9467): 1309–14. 15823380 10.1016/S0140-6736(05)61027-6

[22] Liberati A, Altman DG, Tetzlaff J, Mulrow C, Gøtzsche PC, Ioannidis JP, The PRISMA statement for reporting systematic reviews and meta-analyses of studies that evaluate health care interventions: explanation and elaboration. J Clin Epidemiol 2009; 62(10): E1–34. 19631507 10.1016/j.jclinepi.2009.06.006

[23] Joanna Briggs Institute. The Joanna Briggs Institute Critical Appraisal Tools for Use in JBI Systematic Reviews: Checklist for Prevalence Studies. Joanna Briggs Institute, 2020 (https://jbi.global/critical-appraisal-tools)

[24] Barbieri A, Visco-Comandini F, Fegatelli DA, Schepisi C, Russo V, Calo F, Complex trauma, PTSD and complex PTSD in African refugees. Eur J Psychotraumatol 2019; 10(1): 1700621. 31853336 10.1080/20008198.2019.1700621 PMC6913679

[25] de Jong J, Komproe IH, Spinazzola J, van der Kolk BA, Van Ommeren MH. DESNOS in three postconflict settings: assessing cross-cultural construct equivalence. J Trauma Stress 2005; 18(1): 13–21. 16281191 10.1002/jts.20005

[26] Frost R, Hyland P, McCarthy A, Halpin R, Shevlin M, Murphy J. The complexity of trauma exposure and response: profiling PTSD and CPTSD among a refugee sample. Psychol Trauma 2019; 11(2): 165–75. 30346204 10.1037/tra0000408

[27] Grossman ES, Hoffman YSG, Shrira A, Kedar M, Ben-Ezra M, Dinnayi M, Preliminary evidence linking complex-PTSD to insomnia in a sample of Yazidi genocide survivors. Psychiatry Res 2019; 271: 161–6. 30481693 10.1016/j.psychres.2018.11.044

[28] Hecker T, Huber S, Maier T, Maercker A. Differential associations among PTSD and complex PTSD symptoms and traumatic experiences and postmigration difficulties in a culturally diverse refugee sample. J Trauma Stress 2018; 31(6): 795–804. 30431683 10.1002/jts.22342

[29] Hyland P, Ceannt R, Daccache F, Abou Daher R, Sleiman J, Gilmore B, Are posttraumatic stress disorder (PTSD) and complex-PTSD distinguishable within a treatment-seeking sample of Syrian refugees living in Lebanon? Glob Ment Health 2018; 5: e14. 10.1017/gmh.2018.2 PMC5981765 29868234

[30] Mundy SS, Foss SLW, Poulsen S, Hjorthoj C, Carlsson J. Sex differences in trauma exposure and symptomatology in trauma-affected refugees. Psychiatry Res 2020; 293: 113445. 32977049 10.1016/j.psychres.2020.113445

[31] Nickerson A, Cloitre M, Bryant RA, Schnyder U, Morina N, Schick M. The factor structure of complex posttraumatic stress disorder in traumatized refugees. Eur J Psychotraumatol 2016; 7: 33253. 27989268 10.3402/ejpt.v7.33253 PMC5165057

[32] Palic S, Elklit A. Personality dysfunction and complex posttraumatic stress disorder among chronically traumatized bosnian refugees. JNMD 2014; 202(2): 111–8. 10.1097/NMD.0000000000000079 24469522

[33] Rathke H, Poulsen S, Carlsson J, Palic S. PTSD with secondary psychotic features among trauma-affected refugees: The role of torture and depression. Psychiatry Res 2020; 287: 112898. 32179211 10.1016/j.psychres.2020.112898

[34] Silove D, Rees S, Mohsin M, Tam N, Kareth M, Tay AK. Differentiating ICD-11 complex post-traumatic stress disorder from other common mental disorders based on levels of exposure to childhood adversities, the traumas of persecution and postmigration living difficulties among refugees from West Papua. BJPsych Open 2018; 4(5): 361–7.

[35] Tay AK, Rees S, Chen J, Kareth M, Silove D. The structure of post-traumatic stress disorder and complex post-traumatic stress disorder amongst West Papuan refugees. BMC Psychiatry 2015a; 15: 111. 25947101 10.1186/s12888-015-0480-3 PMC4459680

[36] Vang ML, Nielsen SB, Auning-Hansen M, Elklit A. Testing the validity of ICD-11 PTSD and CPTSD among refugees in treatment using latent class analysis. Torture 2019; 29(3): 27–45. 31984942 10.7146/torture.v29i3.115367

[37] Naing L, Winn T, Rusli BN. Practical issues in calculating the sample size for prevalence studies. Arch Orofac Sci 2006; 1: 9–14.

[38] Richardson LK, Frueh BC, Acierno R. Prevalence estimates of combat-related post-traumatic stress disorder: critical review. Aust NZ J Psychiatry 2010; 44(1): 4–19. 10.3109/00048670903393597 PMC2891773 20073563

[39] Jowett S, Karatzias T, Shevlin M, Albert I. Differentiating symptom profiles of ICD-11 PTSD, complex PTSD, and borderline personality disorder: a latent class analysis in a multiply traumatized sample. Personal Disord 2020; 11(1): 36–45. 31259603 10.1037/per0000346

[40] Weathers FW, Litz BT, Keane TM, Palmieri PA, Marx BP, Schnurr PP. *PTSD Checklist for DSM-5 (PCL-5)*. National Center for PTSD, 2013 (https://www.ptsd.va.gov/professional/assessment/adult-sr/ptsd-checklist.asp).

[41] Pelcovitz D, Kolk BVD, Roth S, Mandel F, Kaplan S, Resick P. Development of a criteria set and a structured interview for disorders of extreme stress (SIDES). J Trauma Stress 1997; 10(1): 3–16. 9018674 10.1023/a:1024800212070

[42] Grant BF, Dawson DA, Stinson FS, Chou PS, Kay W, Pickering R. The Alcohol Use Disorder and Associated Disabilities Interview Schedule-IV (AUDADIS-IV): reliability of alcohol consumption, tobacco use, family history of depression and psychiatric diagnostic modules in a general population sample. Drug Alcohol Depend 2003; 71(1): 7–16. 12821201 10.1016/s0376-8716(03)00070-x

[43] Cloitre M, Shevlin M, Brewin CR, Bisson JI, Roberts NP, Maercker A, The International Trauma Questionnaire: development of a self-report measure of ICD-11 PTSD and complex PTSD. Acta Psychiatr Scand 2018; 138(6): 536–46. 30178492 10.1111/acps.12956

[44] Gratz KL, Roemer L. Multidimensional assessment of emotion regulation and dysregulation: development, factor structure, and initial validation of the Difficulties in Emotion Regulation Scale. J Psychopathol Behav Assess 2004; 26(1): 41–54.

[45] Mollica RF, Wyshak G, De Marneffe D, Khuon F, Lavelle J. Indochinese versions of the Hopkins Symptom Checklist-25: a screening instrument for the psychiatric care of refugees. Am J Psychiatry 1987; 144(4): 497–500. 3565621 10.1176/ajp.144.4.497

[46] Wei M, Russell DW, Mallinckrodt B, Vogel DL. The Experiences in Close Relationship Scale (ECR)-Short Form: reliability, validity, and factor structure. J Pers Assess 2007; 88(2): 187–204. 17437384 10.1080/00223890701268041

[47] Luxenberg T, Spinazzola J, Van der Kolk BA. Complex trauma and disorders of extreme stress (DESNOS) diagnosis, part one: assessment. Dir Psychiatry 2001; 21(25): 3–16.

[48] Tay AK, Rees S, Chen J, Kareth M, Mohsin M, Silove D. The Refugee-Mental Health Assessment Package (R-MHAP); rationale, development and first-stage testing amongst West Papuan refugees. Int J Ment Health Syst 2015; 9(1): 1–13.25587353

[49] Foa EB. Posttraumatic diagnostic scale manual. National Computer Systems, 1996.

