# Supplementary material for: Prevalence of complex post-traumatic stress disorder in refugees and asylum seekers: systematic review
Source: BJPsych Open. 2021 Oct 15;7(6):e194. doi: 10.1192/bjo.2021.1013 (PMC8570104; doi:10.1192/bjo.2021.1013)
Supplement: Supplementary file 1 [file S2056472421010139sup001.docx]

**Supplementary Appendix 1 – Systematic Review Search Strategies**

**Database: PsycINFO**

1 refugees/ (5906)

2 refugee*.tw. (8563)

3 asylum seeking/ (549)

4 (asylum adj3 seek*).tw. (1576)

5 "displacement (defense mechanism)"/ (175)

6 displace*.tw. (14736)

7 immigration/ (21972)

8 immigra*.tw. (32194)

9 migra*.tw. (39642)

10 emigra*.tw. (2063)

11 political asylum/ (34)

12 alien*.tw. (11785)

13 foreign*.tw. (23150)

14 exile*.tw. (1283)

15 stateless*.tw. (115)

16 settlement*.tw. (4065)

17 exp conflict/ (115956)

18 conflict*.tw. (128473)

19 war*.tw. (121776)

20 1 or 2 or 3 or 4 or 5 or 6 or 7 or 8 or 9 or 10 or 11 or 12 or 13 or 14 or 15 or 16 or 17 or 18 or 19 (421564)

21 complex ptsd/ (267)

22 complex ptsd.tw. (467)

23 complex post traumatic stress disorder*.tw. (102)

24 complex posttraumatic stress disorder*.tw. (254)

25 cptsd.tw. (163)

26 posttraumatic stress disorder/ (32118)

27 complex.tw. (197557)

28 26 and 27 (1787)

29 desnos/ (16)

30 "disorder* of extreme stress not otherwise specified".tw. (65)

31 disorder* of extreme stress.tw. (110)

32 Disturbance* in Self Organi?ation.tw. (19)

33 epcace.tw. (6)

34 enduring personality change* after catastrophic event*.tw. (1)

35 enduring personality change* after catastrophic experience*.tw. (12)

36 21 or 22 or 23 or 24 or 25 or 28 or 29 or 30 or 31 or 32 or 33 or 34 or 35 (2108)

37 20 and 36 (551)

**Database: Embase**

1 asylum seeker/ (887)

2 (asylum adj3 seek*).tw. (2086)

3 refugee/ (12054)

4 refugee*.tw. (11069)

5 migrant/ (7162)

6 migra*.tw. (453156)

7 immigrant/ (16017)

8 immigra*.tw. (37021)

9 emigrant/ (311)

10 emigra*.tw. (8508)

11 refugee camp/ (625)

12 displace*.tw. (150043)

13 alien*.tw. (8219)

14 foreign*.tw. (89367)

15 exile*.tw. (701)

16 stateless*.tw. (79)

17 settlement*.tw. (12624)

18 exp war/ (29849)

19 war*.tw. (587816)

20 conflict/ (24323)

21 conflict*.tw. (154724)

22 1 or 2 or 3 or 4 or 5 or 6 or 7 or 8 or 9 or 10 or 11 or 12 or 13 or 14 or 15 or 16 or 17 or 18 or 19 or 20 or 21 (1486764)

23 complex ptsd.tw. (239)

24 complex post traumatic stress disorder*.tw. (63)

25 complex posttraumatic stress disorder*.tw. (117)

26 cptsd.tw. (118)

27 posttraumatic stress disorder/ (58425)

28 complex.tw. (1406831)

29 27 and 28 (2688)

30 DESNOS.tw. (40)

31 "disorder* of extreme stress not otherwise specified".tw. (30)

32 disorder* of extreme stress.tw. (56)

33 Disturbance* in Self Organi?ation.tw. (18)

34 epcace.tw. (9)

35 enduring personality change* after catastrophic event*.tw. (2)

36 enduring personality change* after catastrophic experience*.tw. (17)

37 23 or 24 or 25 or 26 or 29 or 30 or 31 or 32 or 33 or 34 or 35 or 36 (2786)

38 22 and 37 (521)

**Database:** **MEDLINE**

1 Refugees/ (9947)

2 refugee*.tw. (10057)

3 displace*.tw. (135217)

4 (asylum adj3 seek*).tw. (1767)

5 "Transients and Migrants"/ (11369)

6 migra*.tw. (357674)

7 transient*.tw. (325543)

8 exp "Emigrants and Immigrants"/ (12027)

9 alien*.tw. (7475)

10 emigra*.tw. (7432)

11 immigra*.tw. (32421)

12 foreign*.tw. (80073)

13 exile*.tw. (617)

14 stateless*.tw. (78)

15 Refugee Camps/ (134)

16 settlement*.tw. (11729)

17 exp Armed Conflicts/ (10253)

18 conflict*.tw. (122159)

19 exp Warfare/ (36497)

20 war*.tw. (422892)

21 1 or 2 or 3 or 4 or 5 or 6 or 7 or 8 or 9 or 10 or 11 or 12 or 13 or 14 or 15 or 16 or 17 or 18 or 19 or 20 (1481108)

22 Stress Disorders, Post-Traumatic/ (31824)

23 complex.tw. (1195631)

24 22 and 23 (1198)

25 complex ptsd.tw. (230)

26 complex post traumatic stress disorder*.tw. (50)

27 complex posttraumatic stress disorder*.tw. (120)

28 cptsd.tw. (140)

29 DESNOS.tw. (34)

30 disorder* of extreme stress.tw. (48)

31 "disorder* of extreme stress not otherwise specified".tw. (28)

32 Disturbance* in Self Organi?ation.tw. (22)

33 epcace.tw. (6)

34 enduring personality change* after catastrophic experience*.tw. (12)

35 enduring personality change* after catastrophic event*.tw. (1)

36 24 or 25 or 26 or 27 or 28 or 29 or 30 or 31 or 32 or 33 or 34 or 35 (1385)

37 21 and 36 (350)

**Database: PTSDpubs**

(((complex ptsd) OR (MAINSUBJECT.EXACT.EXPLODE("Complex PTSD")) OR (complex post traumatic stress disorder) OR (complex posttraumatic stress disorder) OR CPTSD OR DESNOS OR (disorder* of extreme stress) OR ("disorder* of extreme stress not otherwise specified") OR (disturbance* in self organi?ation) OR EPCACE OR (MAINSUBJECT.EXACT.EXPLODE("Enduring Personality Change")) OR (enduring personality change* after catastrophic experience*) OR (enduring personality change* after catastrophic event*)) AND (emigra* OR immigra* OR migra* OR (MAINSUBJECT.EXACT.EXPLODE("Immigrants")) OR (MAINSUBJECT.EXACT.EXPLODE("Emigrants")) OR (MAINSUBJECT.EXACT.EXPLODE("Exiles")) OR (MAINSUBJECT.EXACT.EXPLODE("Migrants")) OR displace* OR (MAINSUBJECT.EXACT.EXPLODE("Displaced Persons")) OR (asylum seek*) OR (MAINSUBJECT.EXACT.EXPLODE("Asylum Seekers")) OR (MAINSUBJECT.EXACT.EXPLODE("Internally Displaced Persons")) OR (MAINSUBJECT.EXACT.EXPLODE("Refugees")) OR refugee* OR settlement* OR (MAINSUBJECT.EXACT.EXPLODE("Stateless Persons")) OR stateless* OR alien* OR exile* OR foreign* OR (MAINSUBJECT.EXACT.EXPLODE("War")) OR war* OR conflict* OR (MAINSUBJECT.EXACT.EXPLODE("Persecution")) OR (MAINSUBJECT.EXACT.EXPLODE("Migration")))) AND PEER(yes)

**Database: CINAHL**

1 (MH "Refugees")

2 refugee*

3 asylum N2 seek*

4 migra*

5 (MH "Residential Mobility+")

6 immigra*

7 (MH "Immigrants")

8 emigra*

9 displace*

10 (MH "Refugee Camps")

11 settlement*

12 alien*

13 foreign*

14 exile*

15 stateless*

16 (MH "War+")

17 war*

18 conflict*

19 S1 OR S2 OR S3 OR S4 OR S5 OR S6 OR S7 OR S8 OR S9 OR S10 OR S11 OR S12 OR S13 OR S14 OR S15 OR S16 OR S17 OR S18

20 (MH "Stress Disorders, Post-Traumatic")

21 "complex ptsd"

22 cptsd

23 "complex post-traumatic stress disorder"

24 "complex posttraumatic stress disorder"

25 DESNOS

26 "disorder* of extreme stress not otherwise specified"

27 "disorder* of extreme stress"

28 "disturbance* in self-organi?ation"

29 EPCACE

30 "enduring personality change* after catastrophic event*"

31 "enduring personality change* after catastrophic experience*"

32 complex

33 S20 AND S32

34 S21 OR S22 OR S23 OR S24 OR S25 OR S26 OR S27 OR S28 OR S29 OR S30 OR S31 OR S33

35 S19 AND S34

**Database: Web of Science**

1 TS=refugee*

2 TS=(asylum near/2 seek*)

3 TS=displace*

4 TS=migra*

5 TS=immigra*

6 TS=emigra*

7 TS=war*

8 TS=conflict*

9 TS=settlement*

10 TS=alien*

11 TS=foreign*

12 TS=exile*

13 TS=stateless*

14 TS="complex PTSD"

15 TS="complex post-traumatic stress disorder*"

16 TS="complex posttraumatic stress disorder*"

17 TS=cptsd

18 TS=desnos

19 TS="disorder* of extreme stress not otherwise specified"

20 TS="disorder* of extreme stress"

21 TS="disturbance* in self organi?ation"

22 TS=epcace

23 TS="Enduring Personality Change* A Catastrophic Event

24 TS="Enduring Personality Change* A Catastrophic Experience*"

25 #13 OR #12 OR #11 OR #10 OR #9 OR #8 OR #7 OR #6 OR #5 OR #4 OR #3 OR #2 OR #1

26 #24 OR #23 OR #22 OR #21 OR #20 OR #19 OR #18 OR #17 OR #16 OR #15 OR #14

27 #26 AND #25

**Supplementary Appendix 2:** Characteristics of included studies.

| **Publication** | **Sample and host country**  **Sample size (n)** | **Duration in host country (mean ± SD)** | **Percentage female (%)** | **Mean age (years) ± standard deviation**  **Age range** | **Measurement tool (other Dx)** | **Diagnostic criteria of additional diagnoses** | **Prevalence of other diagnoses (%)** | **95% Confidence interval** |
| --- | --- | --- | --- | --- | --- | --- | --- | --- |
| Barbieri et al. (2019)^24^ | Treatment-seeking African refugees residing in Italy.  n = 120 | 11.1 months ± 8.9. | 14 (n = 17) | 25.1 ± 6.7  n.a | Six PCL-5^40^ items to measure ICD-11 PTSD symptom clusters. | PCL-5 PTSD: a rating of 2 (moderate) or above on items from Cluster B (≥1 item), Cluster C (≥1 item), Cluster D (≥2) items, and Cluster E (≥2).  ICD-11 PTSD: six PCL-5 items used as PTSD symptom clusters, with ratings of ≥2 and <2 an indicator of symptom presence and absence, respectively. | PTSD (DSM-5): 79% (n = 95)  PTSD (ICD-11): 38% (n = 46) | PTSD (DSM-5): 71.05 to 85.47  PTSD (ICD-11): 30.12 to 47.27 |
| de Jong et al. (2005)^25^ | Ethiopian sample: randomly sampled from temporary refugee shelters in Eritrea.  n = 1200 | n.a | 62.4% (n=759) | 33.9 ± 9.9  n.a | n.a. | n.a. | n.a. | n.a. |
| Frost et al. (2019)^26^ | Refugees living in the United States.  n = 308 | n.a.  46.8% reported refugee status lasting over 2 years. | 48.7 (n = 150) | 50.94 ± 17.07  21-45 years | Items from the PTSD scale of the AUDADIS-IV^42^ as indicators of ICD-11 PTSD. | Selected items were coded as present (1) or absent (0).  PTSD: one of two symptoms are endorsed from every PTSD symptom cluster. | PTSD (ICD-11): 20.9% (n = 65) | 16.92 to 26.00 |
| Grossman et al. (2019)^27^ | Female Yazidi captives one to four months following liberation from ISIS, released/resettled in post-ISIS camps in Kurdistan.  n = 108 | n.a.  1-4 months post-liberation. | 100 (n = 108) | 24.41 ±  5.71  n.a. | ITQ^14^ | PTSD: one of two symptoms in every PTSD cluster endorsed, indicated by symptom item scores of 2 or 3. | PTSD (ICD-11): 21.3% (n = 23) | 14.63 to 29.93 |
| Hecker et al. (2018)^28^ | Help- and advice-seeking refugees resettled in Switzerland.  n = 94 | n.a. | 14.9 (n = 14) | 31.60 ± 10.14  18-61 years | ITQ^43^ | PTSD: at least 1 item in every PTSD cluster is endorsed (rated 2 (moderately) or above) with the additional functional impairment fulfilled. | PTSD (ICD-11): 32.9% (n = 31) | 24.31 to 42.99 |
| Hyland et al. (2018)^29^ | Treatment-seeking Syrian refugees living in Lebanon.  n = 110 | 37.45 months ± 14.62. | 80.2 (n = 88) | 33.02 ± 8.94  n.a. | ITQ (M. Cloitre, personal communication, 27 September 2021). | PTSD: the endorsement of at least one symptom from every PTSD cluster, and one of three items assessing functional impairment. | PTSD (ICD-11): 25.2% (n = 27) | 17.45 to 33.36 |
| Liddell et al. (2019)^4^ | Treatment- or help-seeking refugees resettled in Sydney, Australia.  n = 112 | n.a. | 33.9 (n = 38) | 37.7 ± 11.5  18-70 years | Six items reflecting ICD-11 PTSD symptom clusters from the PSS-I.^50^ | Meeting the specified clinical thresholds for every symptom clusters. | PTSD (ICD-11) 23.5% (n = 26) | 16.36 to 31.84 |
| Mundy et al. (2020)^30^ | Treatment-seeking refugees in Denmark with a diagnosis of PTSD.  n = 318 | 15 years ± 8.6. | 52.8 (n = 168) | 45 ± 10.3  19-70 years | PTSD: ICD-10 PTSD criteria^9^  Depression: ICD-10 criteria for recurrent depression or depressive episodes.^9^ | The fulfilment of ICD-10 criteria, assessed through clinical interviews pre-treatment. | PTSD (ICD-10): 100% (n = 318)  Depression (recurrent or single episode): 93.8% (n = 213 from n = 227)  Other: 33.2% (n = 62) | PTSD: 98.81 to 100  Depression (ICD-10): 89.92 to 96.29  Other: 15.52 to 24.20 |
| Nickerson et al. (2016)^31^ | Treatment-seeking refugees and asylum-seekers resettled in Switzerland.  n = 134 | 9.01 years ± 6.67. | 21.6 (n = 29) | 42.4 ± 9.8  n.a. | Six items from the PDS,^49^ with two representing each PTSD symptom cluster. | PTSD: endorsing at least one item from every PTSD symptom cluster, and not fulfilling complex PTSD criteria. | 19.7% (n = 26) | 13.60 to 26.91 |
| Palic & Elklit (2014)^32^ | Treatment-seeking Bosnian former refugees resettled in Denmark.  n = 116 | 16.1 years ± 3.0 | 53 (n = 61) | 46.5 ± 8.1  30-67 years | n.a. | n.a. | n.a. | n.a. |
| Rathke et al. (2020)^33^ | Treatment-seeking refugees resettled in Denmark.  n = 627 | Total sample: 14.9 years  Complex PTSD sample: n.a. | Total sample: 41.8 (n = 262)  Complex PTSD: n.a. | n.a | PTSD: ICD-10 PTSD criteria.^9^  Psychotic symptoms: SCAN.^51^  PTSD with secondary psychotic features (PTSD-SP): SCAN.^51^  Depression: HAM-D.^52^ | PTSD diagnoses made by medical doctors during routine assessments of ICD-10 criteria, often facilitated by the initial 16 questions of the HTQ (part 4).  PTSD-SP: meeting PTSD criteria in addition to one or more psychotic symptom from the following: persecutory delusions, visual hallucinations, and auditory hallucinations.  Depression: binary scores were recorded with a score of 20 taken as the cut-off for clinically relevant depressive symptoms. | - PTSD only: 2.6% (n = 16 from n = 624) - PTSD and comorbid EPCACE: 49.5% (n = 165 from n = 333) - PTSD-SP and comorbid EPCACE: 43.9% (n = 47 from n = 107 - PTSD and comorbid depression: 25.8% (n = 148 from n = 573) - PTSD-SP and comorbid depression: 25.8% (n = 148 from n = 573) - PTSD-SP without comorbid depression: 4.2% (n = 24 from n = 573) | - PTSD only: 1.58 to 4.10 - PTSD and comorbid EPCACE: 44.22 to 54.89 - PTSD-SP and comorbid EPCACE: 34.89 to 53.38 - PTSD and comorbid depression: 22.41 to 29.57 - PTSD-SP and comorbid depression: 22.41 to 29.57 - PTSD-SP without comorbid depression: 2.83 to 6.16 |
| Silove et al. (2018)^34^ | West Papuan refugees residing in PNG, from 9 identified settlements.  n = 487 | 15.57 years ± .48 | 44.1 (n = 215) | 35.81 ± .65  n.a. | R-MHAP^48^ for CMDs prevalent among refugees. | Algorithms derived from definitions of disorders in the ICD-10, ICD-11, DSM-4 and DSM-5 were used to provide diagnoses. Symptom endorsement requires the highest two ratings from a four-point severity scale. | - PTSD (DSM-5): 12.1% (n = 59) - MDD: 56.2%   (n = 273)   - GAD: 26.5%   (n = 129)   - Panic disorder: 8% (n = 39) - Persistent complicated bereavement disorder: 9.9%   (n = 48)   - Separation anxiety disorder: 9.9% (n = 48) - Intermittent explosive disorder: 5.6%   (n = 27) | - PTSD (DSM-5): 9.51 to 15.31 - MDD: 51.62 to 60.4 - GAD: 22.76 to 30.58 - Panic disorder: 5.91 to 10.76 - Persistent complicated bereavement disorder: 7.51 to 12.83 - Separation anxiety disorder: 7.51 to 12.83 - Intermittent explosive disorder: 3.84 to 7.95 |
| Tay et al. (2015a)^35^ | West-Papuan refugees residing in settlements in Port Moresby, Papua New Guinea.  n = 230 | West Papuan born participants: 27 years ± 10.28. | 40.4 (n = 93) | 37 ± 9.80  n.a. | R-MHAP^48^ | Algorithms derived from definitions of disorders in the ICD-10, ICD-11, DSM-4, and DSM-5 were used to provide diagnoses. | - PTSD (DSM-4): 13% (n = 30) - PTSD (DSM-5): 12% (n = 28) - PTSD (ICD-10): 13% (n = 30) - PTSD (ICD-11): 6% (n = 14) | - PTSD (DSM-4): 9.29 to 18.01 - PTSD (DSM-5): 8.56 to 17.03 - PTSD (ICD-10): 9.29 to 18.01 - PTSD (ICD-11): 3.66 to 9.96 |
| Teodorescu et al. (2012)^3^ | Treatment-seeking refugees resettled in Norway.  n = 61 | 15.8 years ± 6.4. | 41 (n = 25) | Male: 44  Female: 38  n.a. | PTSD: SCID-PTSD^53^  Axis 1 DSM-IV conditions:  MINI^54^ | PTSD: the fulfilment of all six SCID-PTSD criteria  MINI: fulfilment of the specified criteria for axis 1 DSM-4 disorders | - PTSD: 82% (n = 50) - MDD: 71% (n = 43) - Dysthymia: 49% (n = 30) - Hypomania: 0% (n = 0) - Panic disorder: 41% (n = 5) - Agoraphobia: 49% (n = 30) - Social phobia: 49% (n = 30) - OCD: 44% (n = 27) - GAD: 31% (n = 19) - Alcohol abuse disorder: 10% (n = 6) - Nicotine dependence: 36% (n = 20) - Bulimia: 5% (n = 3) | - PTSD: 70.53 to 89.62 - MDD: 58.11 to 80.44 - Dysthymia: 37.06 to 61.40 - Hypomania: 0 to 5.92 - Panic disorder: 3.55 to 17.79 - Agoraphobia: 37.06 to 61.40 - Social phobia: 37.06 to 61.40 - OCD: 32.51 to 56.70 - GAD: 20.94 to 43.59 - Alcohol abuse disorder: 4.59 to 19.84 - Nicotine dependence: 22.34 to 45.28 - Bulimia: 1.69 to 13.49 |
| Vang et al. (2019)^36^ | Treatment-seeking refugees residing in Denmark.  n = 284 | n.a.  Median arrival year = 2011. | 47.5 (n = 135) | 40.94 ± 9.77  17-68 years | ITQ^43^ | PTSD: one of two symptoms in each PTSD cluster endorsed, indicated by symptom item scores of 2 or 3. | 25% (n = 71) | 20.32 to 30.35 |

^50^PTSD Symptom Scale-Interview version (PSS-I); ^51^Schedules for Clinical Assessment in Neuropsychiatry (SCAN); ^52^Hamilton Depression Scale (HAM-D); ^53^Structured Clinical Interview for DSM-IV-TR PTSD Module (SCID-PTSD); ^54^Mini-International Neuropsychiatric Interview 5.0.0 (MINI).

**References:**

**50 Foa EB, Cashman L, Jaycox L, Perry K.** The validation of a self-report measure of posttraumatic stress disorder: The Posttraumatic Diagnostic Scale. *Psychol Assess* 1997; **9**(4): 445–451.

**51 World Health Organisation.** *Schedules for clinical assessment in neuropsychiatry*. WHO, 1994.

**52 Hamilton M. A rating scale for depression.** *J Neurol Neurosurg Psychiatry* 1969; **23**(1): 56-62.

**53 First MB, Spitzer RL, Gibbon M, Williams JWB.** *Structured Clinical Interview for DSM-IV-TR Axis I Disorders-Patient Edition (SCID-I/P, 1/2007 revision).* Biometrics Research Department New York State Psychiatric Institute, 2007.

**54 Sheehan DV, Lecrubier Y, Sheehan KH, Amorim P, Janavs J, Weiller E, et al.** The Mini-International Neuropsychiatric Interview (M.I.N.I.): The development and validation of a structured diagnostic psychiatric interview for DSM-IV and ICD-10. *J Clin Psychiatry* 1998; **59**(Suppl 20), 22–33.

**Supplementary Appendix 3: PRISMA 2009 Checklist**

| **Section/topic** | **#** | **Checklist item** | **Reported on page #** |
| --- | --- | --- | --- |
| **TITLE** | | |  |
| Title | 1 | Identify the report as a systematic review, meta-analysis, or both. | 1 |
| **ABSTRACT** | | |  |
| Structured summary | 2 | Provide a structured summary including, as applicable: background; objectives; data sources; study eligibility criteria, participants, and interventions; study appraisal and synthesis methods; results; limitations; conclusions and implications of key findings; systematic review registration number. | 1 |
| **INTRODUCTION** | | |  |
| Rationale | 3 | Describe the rationale for the review in the context of what is already known. | 1-3 |
| Objectives | 4 | Provide an explicit statement of questions being addressed with reference to participants, interventions, comparisons, outcomes, and study design (PICOS). | 3 |
| **METHODS** | | |  |
| Protocol and registration | 5 | Indicate if a review protocol exists, if and where it can be accessed (e.g., Web address), and, if available, provide registration information including registration number. | 3 |
| Eligibility criteria | 6 | Specify study characteristics (e.g., PICOS, length of follow-up) and report characteristics (e.g., years considered, language, publication status) used as criteria for eligibility, giving rationale. | 3 |
| Information sources | 7 | Describe all information sources (e.g., databases with dates of coverage, contact with study authors to identify additional studies) in the search and date last searched. | 3 |
| Search | 8 | Present full electronic search strategy for at least one database, including any limits used, such that it could be repeated. | Supplementary Appendix 1 |
| Study selection | 9 | State the process for selecting studies (i.e., screening, eligibility, included in systematic review, and, if applicable, included in the meta-analysis). | 3 |
| Data collection process | 10 | Describe method of data extraction from reports (e.g., piloted forms, independently, in duplicate) and any processes for obtaining and confirming data from investigators. | 3 |
| Data items | 11 | List and define all variables for which data were sought (e.g., PICOS, funding sources) and any assumptions and simplifications made. | 3 |
| Risk of bias in individual studies | 12 | Describe methods used for assessing risk of bias of individual studies (including specification of whether this was done at the study or outcome level), and how this information is to be used in any data synthesis. | 3 |
| Summary measures | 13 | State the principal summary measures (e.g., risk ratio, difference in means). | 3 |
| Synthesis of results | 14 | Describe the methods of handling data and combining results of studies, if done, including measures of consistency (e.g., I^2^) for each meta-analysis. | None |
| Risk of bias across studies | 15 | Specify any assessment of risk of bias that may affect the cumulative evidence (e.g., publication bias, selective reporting within studies). | None |
| Additional analyses | 16 | Describe methods of additional analyses (e.g., sensitivity or subgroup analyses, meta-regression), if done, indicating which were pre-specified. | None |
| **RESULTS** | | |  |
| Study selection | 17 | Give numbers of studies screened, assessed for eligibility, and included in the review, with reasons for exclusions at each stage, ideally with a flow diagram. | 4 |
| Study characteristics | 18 | For each study, present characteristics for which data were extracted (e.g., study size, PICOS, follow-up period) and provide the citations. | 6, Supplementary Appendix 2 |
| Risk of bias within studies | 19 | Present data on risk of bias of each study and, if available, any outcome level assessment (see item 12). | 9 |
| Results of individual studies | 20 | For all outcomes considered (benefits or harms), present, for each study: (a) simple summary data for each intervention group (b) effect estimates and confidence intervals, ideally with a forest plot. | 5, 8 |
| Synthesis of results | 21 | Present results of each meta-analysis done, including confidence intervals and measures of consistency. | None |
| Risk of bias across studies | 22 | Present results of any assessment of risk of bias across studies (see Item 15). | None |
| Additional analysis | 23 | Give results of additional analyses, if done (e.g., sensitivity or subgroup analyses, meta-regression [see Item 16]). | None |
| **DISCUSSION** | | |  |
| Summary of evidence | 24 | Summarize the main findings including the strength of evidence for each main outcome; consider their relevance to key groups (e.g., healthcare providers, users, and policy makers). | 7-8 |
| Limitations | 25 | Discuss limitations at study and outcome level (e.g., risk of bias), and at review-level (e.g., incomplete retrieval of identified research, reporting bias). | 8-9 |
| Conclusions | 26 | Provide a general interpretation of the results in the context of other evidence, and implications for future research. | 9-10 |
| **FUNDING** | | |  |
| Funding | 27 | Describe sources of funding for the systematic review and other support (e.g., supply of data); role of funders for the systematic review. | None |
